# 2-*N*-Benzyl-2,6-dide­oxy-2,6-imino-3,4-*O*-iso­propyl­idene-d-allono­nitrile

**DOI:** 10.1107/S1600536813030584

**Published:** 2013-11-13

**Authors:** Benjamin J. Ayers, Sarah F. Jenkinson, George W. J. Fleet, Amber L. Thompson

**Affiliations:** a Department of Organic Chemistry, Chemistry Research Laboratory, University of Oxford, Oxford OX1 3TA, England; b Department of Chemical Crystallography, Chemistry Research Laboratory, University of Oxford, Oxford OX1 3TA, England

## Abstract

X-ray crystallography firmly established the relative stereochemistry of the title compound, C_16_H_20_N_2_O_3_. The acetonide ring adopts an envelope conformation with one of the O atoms as the flap and the piperidine ring adopts a slightly twisted boat conformation. The absolute configuration was determined by use of d-ribose as the starting material. The compound exists as O—H⋯O hydrogen-bonded chains of mol­ecules running parallel to the *b* axis.

## Related literature

For the biological activity of polyhy­droxy­lated piperidines, see: Nash *et al.* (2011 ▶); Watson *et al.* (2001 ▶). For a related α-imino­nitrile, see: Ayers *et al.* (2012 ▶). For the hydrogen-atom treatment, see; Cooper *et al.* (2010 ▶). For details of the low temperature equipment used in the experiment, see: Cosier & Glazer (1986 ▶). For the weighting scheme, see: Prince (1982 ▶); Watkin (1994 ▶). 
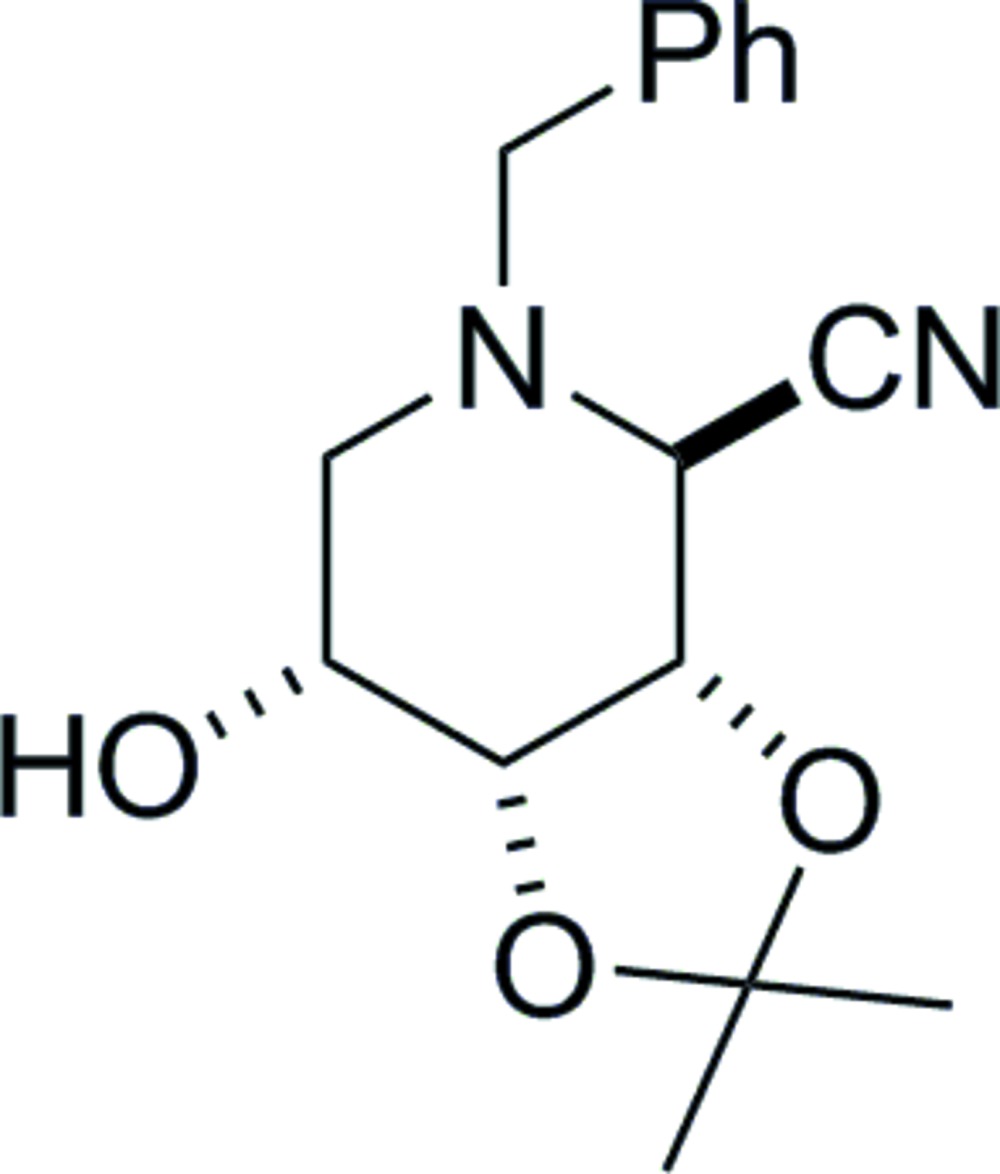


## Experimental

### 

#### Crystal data

C_16_H_20_N_2_O_3_
*M* *_r_* = 288.35
Orthorhombic, 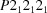

*a* = 8.3978 (3) Å
*b* = 11.2689 (4) Å
*c* = 15.9210 (6) Å
*V* = 1506.67 (9) Å^3^
*Z* = 4
Mo *K*α radiation
μ = 0.09 mm^−1^
*T* = 150 K
0.4 × 0.4 × 0.2 mm

#### Data collection

Nonius KappaCCD diffractometer
Absorption correction: multi-scan (*DENZO*/*SCALEPACK*; Otwinowski & Minor, 1997 ▶) *T*_min_ = 0.91, *T*_max_ = 0.98
11529 measured reflections
1970 independent reflections
1422 reflections with *I* > 2.0σ(*I*)
*R*_int_ = 0.079

#### Refinement

*R*[*F* ^2^ > 2σ(*F* ^2^)] = 0.044
*wR*(*F* ^2^) = 0.121
*S* = 0.95
1970 reflections
190 parameters
H-atom parameters constrained
Δρ_max_ = 0.47 e Å^−3^
Δρ_min_ = −0.42 e Å^−3^

### 

Data collection: *COLLECT* (Nonius, 2001 ▶).; cell refinement: *DENZO*/*SCALEPACK* (Otwinowski & Minor, 1997 ▶); data reduction: *DENZO*/*SCALEPACK*; program(s) used to solve structure: *SIR92* (Altomare *et al.*, 1994 ▶); program(s) used to refine structure: *CRYSTALS* (Betteridge *et al.*, 2003 ▶); molecular graphics: *CAMERON* (Watkin *et al.*, 1996 ▶); software used to prepare material for publication: *CRYSTALS*.

## Supplementary Material

Crystal structure: contains datablock(s) I, global. DOI: 10.1107/S1600536813030584/lh5665sup1.cif

Structure factors: contains datablock(s) I. DOI: 10.1107/S1600536813030584/lh5665Isup2.hkl

Additional supplementary materials:  crystallographic information; 3D view; checkCIF report

## Figures and Tables

**Table 1:** Hydrogen-bond geometry (Å, °)

*D*—H⋯*A*	*D*—H	H⋯*A*	*D*⋯*A*	*D*—H⋯*A*
O1—H11⋯O6^i^	0.86	2.05	2.850 (5)	156 (1)

Symmetry code: (i) 
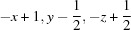
.

**Fig. 1.**
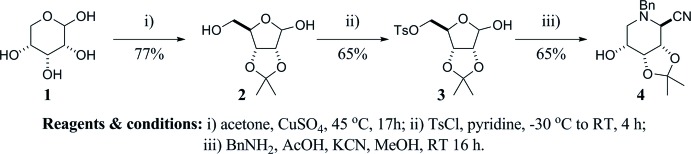
Synthetic Scheme

**Fig. 2.**
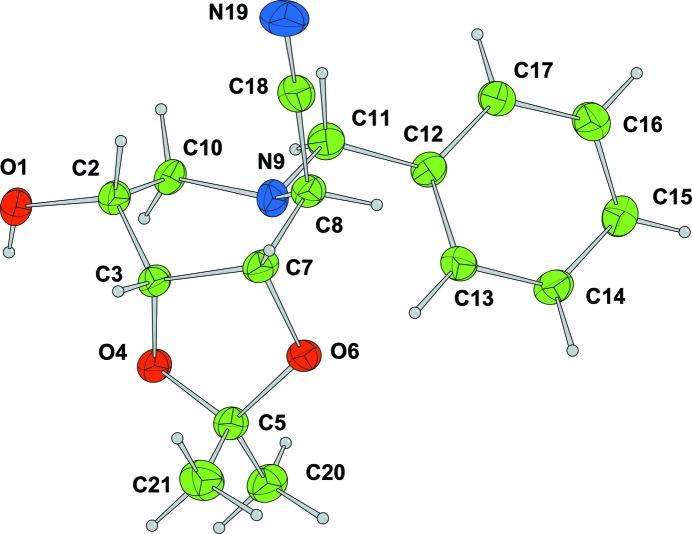
The title compound with displacement ellipsoids drawn at the 50% probability level. H atoms are shown as spheres of arbitary radius.

**Fig. 3.**
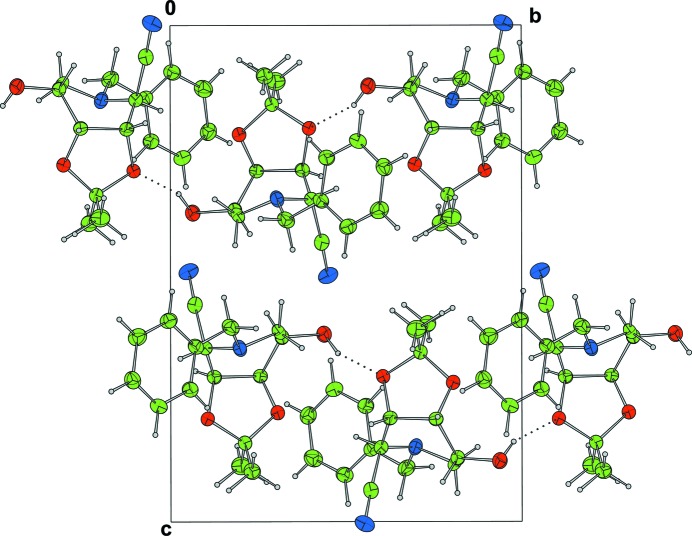
Packing diagram for the crystal projected along the a-axis. Hydrogen bonds are shown as dotted lines.

## supplementary crystallographic information

### 1. Comment

Many polyhydroxylated piperidines have been found to display interesting biological properties (Nash *et al.*, 2011; Watson *et al.*, 2001). Piperidine α-iminonitrile 4 was prepared from 2,3-*O*-isopropylidene-5-*O*-toluenesulfonyl-*D*-ribose 3 by a tandem Strecker reaction and iminocyclization (Fig. 1). The title crystal structure establishes the relative configuration of 4. The absolute configuration is determined by use of D-ribose 1 as the starting material. The acetonide ring adopts an envelope conformation with O4 out of the plane and the piperidine ring adopts a slightly twisted boat conformation with the nitrile group in the flagpole position (Fig. 2). The compound exists as O—H···O hydrogen bonded chains of molecules running parallel to the *b*-axis (Fig. 3). Only classical hydrogen bonding was considered.

### 2. Experimental

α-Iminonitrile 4 was recrystallized by diffusion from a mixture of ethyl acetate and cyclohexane: m.p. 342–344 K; [α]_D_^20^ +20.3 (*c* 1.75, methanol).

### 3. Refinement

In the absence of significant anomalous scattering, Friedel pairs were merged and the absolute configuration was assigned from the use of D-ribose as the starting material.

The H atoms were all located in a difference map, but those attached to carbon atoms were repositioned geometrically. The H atoms were initially refined with soft restraints on the bond lengths and angles to regularize their geometry (C—H in the range 0.93–0.98, N—H in the range 0.86–0.89 N—H to 0.86 O—H = 0.82 Å) and *U*_iso_(H) (in the range 1.2–1.5 times *U*_eq_ of the parent atom), after which the positions were refined with riding constraints (Cooper *et al.*, 2010).

### Crystal data

**Table:**

C_16_H_20_N_2_O_3_	*F*(000) = 616
*M**_r_* = 288.35	*D*_x_ = 1.271 Mg m^−^^3^
Orthorhombic, *P*2_1_2_1_2_1_	Mo *K*α radiation, λ = 0.71073 Å
Hall symbol: P 2ac 2ab	Cell parameters from 1934 reflections
*a* = 8.3978 (3) Å	θ = 5–27°
*b* = 11.2689 (4) Å	µ = 0.09 mm^−^^1^
*c* = 15.9210 (6) Å	*T* = 150 K
*V* = 1506.67 (9) Å^3^	Plate, colourless
*Z* = 4	0.4 × 0.4 × 0.2 mm

### Data collection

**Table:**

Nonius KappaCCD diffractometer	1422 reflections with *I* > 2.0σ(*I*)
Graphite monochromator	*R*_int_ = 0.079
ω scans	θ_max_ = 27.5°, θ_min_ = 5.1°
Absorption correction: multi-scan (*DENZO*/*SCALEPACK*; Otwinowski & Minor, 1997)	*h* = −10→10
*T*_min_ = 0.91, *T*_max_ = 0.98	*k* = −14→14
11529 measured reflections	*l* = −20→20
1970 independent reflections	

### Refinement

**Table:**

Refinement on *F*^2^	Primary atom site location: structure-invariant direct methods
Least-squares matrix: full	Hydrogen site location: difference Fourier map
*R*[*F*^2^ > 2σ(*F*^2^)] = 0.044	H-atom parameters constrained
*wR*(*F*^2^) = 0.121	Method, part 1, Chebychev polynomial, (Watkin, 1994, *P*rince, 1982) [*w*eight] = 1.0/[A_0_*T_0_(x) + A_1_*T_1_(x) ··· + A_n-1_]*T_n-1_(x)] where A_i_ are the Chebychev coefficients listed belo*w* and x = *F* /*F*max Method = Robust Weighting (*P*rince, 1982) W = [*w*eight] * [1-(delta*F*/6*sigma*F*)^2^]^2^ A_i_ are: 9.51 13.9 7.19 2.01
*S* = 0.95	(Δ/σ)_max_ = 0.0000939
1970 reflections	Δρ_max_ = 0.47 e Å^−^^3^
190 parameters	Δρ_min_ = −0.42 e Å^−^^3^
0 restraints	

### Special details

**Table:**

Experimental. The crystal was placed in the cold stream of an Oxford Cryosystems open-flow nitrogen cryostat (Cosier & Glazer, 1986) with a nominal stability of 0.1 K.

### Fractional atomic coordinates and isotropic or equivalent isotropic displacement parameters (Å^2^)

**Table:**

	*x*	*y*	*z*	*U*_iso_*/*U*_eq_	
O1	0.6465 (3)	0.06340 (19)	0.37776 (14)	0.0305	
C2	0.5963 (4)	0.1832 (3)	0.37195 (18)	0.0259	
C3	0.6544 (4)	0.2450 (3)	0.29350 (18)	0.0245	
O4	0.5816 (3)	0.19584 (18)	0.21979 (12)	0.0272	
C5	0.5779 (4)	0.2890 (3)	0.15876 (18)	0.0261	
O6	0.5489 (3)	0.39518 (18)	0.20739 (13)	0.0271	
C7	0.6006 (4)	0.3759 (3)	0.29150 (18)	0.0256	
C8	0.4595 (4)	0.4018 (3)	0.35050 (19)	0.0263	
N9	0.3478 (3)	0.3035 (2)	0.34958 (16)	0.0261	
C10	0.4160 (4)	0.1919 (3)	0.38247 (19)	0.0270	
C11	0.1968 (4)	0.3292 (3)	0.3920 (2)	0.0318	
C12	0.1021 (4)	0.4285 (3)	0.35313 (19)	0.0274	
C13	0.0911 (5)	0.4450 (3)	0.26682 (19)	0.0315	
C14	−0.0032 (5)	0.5344 (3)	0.23328 (19)	0.0323	
C15	−0.0876 (4)	0.6088 (3)	0.2858 (2)	0.0308	
C16	−0.0756 (5)	0.5947 (3)	0.3724 (2)	0.0343	
C17	0.0182 (4)	0.5058 (3)	0.4056 (2)	0.0292	
C18	0.5178 (5)	0.4307 (3)	0.4370 (2)	0.0328	
N19	0.5612 (5)	0.4487 (3)	0.50421 (18)	0.0470	
C20	0.4377 (4)	0.2689 (3)	0.1017 (2)	0.0328	
C21	0.7352 (5)	0.3002 (4)	0.1127 (2)	0.0385	
H21	0.6469	0.2260	0.4201	0.0308*	
H31	0.7717	0.2399	0.2892	0.0303*	
H71	0.6919	0.4291	0.3037	0.0311*	
H81	0.4052	0.4724	0.3289	0.0310*	
H101	0.3912	0.1842	0.4427	0.0319*	
H102	0.3691	0.1265	0.3501	0.0317*	
H111	0.2187	0.3478	0.4519	0.0382*	
H112	0.1318	0.2571	0.3895	0.0380*	
H131	0.1502	0.3963	0.2302	0.0381*	
H141	−0.0094	0.5442	0.1754	0.0379*	
H151	−0.1536	0.6691	0.2632	0.0372*	
H161	−0.1325	0.6461	0.4082	0.0410*	
H171	0.0255	0.4957	0.4642	0.0353*	
H201	0.4276	0.3351	0.0634	0.0489*	
H202	0.3408	0.2637	0.1346	0.0490*	
H203	0.4527	0.1972	0.0698	0.0487*	
H212	0.7322	0.3683	0.0748	0.0564*	
H213	0.8215	0.3108	0.1527	0.0572*	
H211	0.7541	0.2275	0.0804	0.0572*	
H11	0.5995	0.0236	0.3393	0.0472*	

### Atomic displacement parameters (Å^2^)

**Table:**

	*U*^11^	*U*^22^	*U*^33^	*U*^12^	*U*^13^	*U*^23^
O1	0.0390 (13)	0.0236 (11)	0.0289 (11)	0.0062 (10)	−0.0064 (10)	0.0008 (9)
C2	0.0334 (17)	0.0206 (14)	0.0237 (14)	0.0016 (14)	−0.0040 (13)	0.0000 (12)
C3	0.0305 (15)	0.0222 (13)	0.0208 (13)	0.0006 (14)	−0.0048 (14)	0.0002 (12)
O4	0.0383 (13)	0.0206 (9)	0.0227 (10)	−0.0004 (11)	−0.0020 (10)	0.0007 (9)
C5	0.0354 (17)	0.0203 (13)	0.0225 (14)	−0.0022 (14)	0.0008 (14)	0.0010 (12)
O6	0.0408 (13)	0.0194 (9)	0.0210 (10)	−0.0008 (10)	−0.0012 (10)	−0.0008 (8)
C7	0.0325 (16)	0.0229 (14)	0.0214 (14)	−0.0036 (13)	−0.0041 (14)	0.0010 (12)
C8	0.0354 (18)	0.0217 (14)	0.0218 (13)	−0.0011 (14)	−0.0012 (13)	0.0010 (12)
N9	0.0293 (13)	0.0201 (12)	0.0289 (12)	0.0013 (11)	0.0032 (12)	0.0036 (11)
C10	0.0359 (17)	0.0205 (14)	0.0247 (14)	0.0011 (14)	0.0014 (14)	0.0061 (12)
C11	0.0353 (19)	0.0314 (17)	0.0285 (16)	0.0043 (15)	0.0085 (15)	0.0053 (14)
C12	0.0306 (17)	0.0264 (15)	0.0253 (14)	−0.0003 (14)	0.0030 (13)	0.0033 (13)
C13	0.0408 (19)	0.0280 (15)	0.0257 (14)	0.0045 (16)	0.0066 (15)	0.0009 (13)
C14	0.042 (2)	0.0315 (16)	0.0236 (15)	−0.0002 (16)	−0.0002 (15)	0.0040 (13)
C15	0.0306 (17)	0.0283 (15)	0.0336 (16)	0.0003 (15)	−0.0043 (15)	0.0030 (14)
C16	0.0340 (19)	0.0366 (18)	0.0323 (16)	0.0076 (16)	−0.0012 (15)	−0.0040 (14)
C17	0.0304 (18)	0.0315 (17)	0.0258 (15)	0.0027 (14)	0.0001 (14)	−0.0024 (13)
C18	0.046 (2)	0.0235 (15)	0.0292 (16)	0.0004 (16)	0.0010 (15)	−0.0013 (13)
N19	0.072 (3)	0.0403 (17)	0.0285 (15)	−0.0022 (19)	−0.0039 (16)	−0.0078 (13)
C20	0.0394 (19)	0.0299 (17)	0.0289 (16)	−0.0040 (15)	−0.0061 (16)	0.0013 (13)
C21	0.040 (2)	0.0405 (19)	0.0349 (19)	0.0005 (18)	0.0091 (16)	0.0004 (18)

### Geometric parameters (Å, º)

**Table:**

O1—C2	1.418 (4)	C11—C12	1.506 (5)
O1—H11	0.855	C11—H111	0.993
C2—C3	1.511 (4)	C11—H112	0.980
C2—C10	1.527 (5)	C12—C13	1.390 (4)
C2—H21	1.000	C12—C17	1.397 (4)
C3—O4	1.434 (4)	C13—C14	1.388 (5)
C3—C7	1.543 (4)	C13—H131	0.942
C3—H31	0.990	C14—C15	1.381 (5)
O4—C5	1.431 (3)	C14—H141	0.929
C5—O6	1.446 (4)	C15—C16	1.392 (5)
C5—C20	1.504 (5)	C15—H151	0.948
C5—C21	1.516 (5)	C16—C17	1.379 (5)
O6—C7	1.424 (3)	C16—H161	0.943
C7—C8	1.540 (5)	C17—H171	0.943
C7—H71	0.992	C18—N19	1.148 (4)
C8—N9	1.452 (4)	C20—H201	0.968
C8—C18	1.498 (4)	C20—H202	0.970
C8—H81	0.980	C20—H203	0.963
N9—C10	1.477 (4)	C21—H212	0.976
N9—C11	1.466 (4)	C21—H213	0.972
C10—H101	0.986	C21—H211	0.981
C10—H102	0.982		
			
C2—O1—H11	108.4	N9—C10—H102	107.3
O1—C2—C3	113.4 (3)	H101—C10—H102	111.1
O1—C2—C10	110.4 (3)	N9—C11—C12	114.5 (3)
C3—C2—C10	112.4 (3)	N9—C11—H111	108.9
O1—C2—H21	106.4	C12—C11—H111	109.6
C3—C2—H21	105.9	N9—C11—H112	107.4
C10—C2—H21	107.8	C12—C11—H112	107.8
C2—C3—O4	111.2 (2)	H111—C11—H112	108.5
C2—C3—C7	111.3 (3)	C11—C12—C13	122.8 (3)
O4—C3—C7	103.1 (2)	C11—C12—C17	118.9 (3)
C2—C3—H31	110.6	C13—C12—C17	118.3 (3)
O4—C3—H31	110.2	C12—C13—C14	121.0 (3)
C7—C3—H31	110.2	C12—C13—H131	119.9
C3—O4—C5	106.4 (2)	C14—C13—H131	119.1
O4—C5—O6	104.3 (2)	C13—C14—C15	120.0 (3)
O4—C5—C20	108.4 (3)	C13—C14—H141	120.0
O6—C5—C20	108.4 (3)	C15—C14—H141	120.0
O4—C5—C21	111.8 (3)	C14—C15—C16	119.6 (3)
O6—C5—C21	109.7 (3)	C14—C15—H151	120.3
C20—C5—C21	113.8 (3)	C16—C15—H151	120.1
C5—O6—C7	109.0 (2)	C15—C16—C17	120.3 (3)
C3—C7—O6	104.8 (2)	C15—C16—H161	119.4
C3—C7—C8	113.2 (3)	C17—C16—H161	120.3
O6—C7—C8	108.1 (3)	C12—C17—C16	120.8 (3)
C3—C7—H71	110.3	C12—C17—H171	118.9
O6—C7—H71	109.1	C16—C17—H171	120.3
C8—C7—H71	111.1	C8—C18—N19	177.4 (4)
C7—C8—N9	110.3 (2)	C5—C20—H201	109.5
C7—C8—C18	110.5 (3)	C5—C20—H202	109.9
N9—C8—C18	112.8 (3)	H201—C20—H202	108.3
C7—C8—H81	107.4	C5—C20—H203	110.1
N9—C8—H81	108.4	H201—C20—H203	109.0
C18—C8—H81	107.3	H202—C20—H203	110.1
C8—N9—C10	113.3 (3)	C5—C21—H212	110.0
C8—N9—C11	113.8 (3)	C5—C21—H213	110.1
C10—N9—C11	109.9 (2)	H212—C21—H213	109.1
C2—C10—N9	113.6 (3)	C5—C21—H211	109.0
C2—C10—H101	108.1	H212—C21—H211	109.6
N9—C10—H101	109.8	H213—C21—H211	109.0
C2—C10—H102	107.0		

### Hydrogen-bond geometry (Å, º)

**Table:**

*D*—H···*A*	*D*—H	H···*A*	*D*···*A*	*D*—H···*A*
O1—H11···O6^i^	0.86	2.05	2.850 (5)	156 (1)

Symmetry code: (i) −*x*+1, *y*−1/2, −*z*+1/2.
